# Nanostructure driven innovation in diabetes: from enhanced biosensing to smart therapeutic delivery

**DOI:** 10.3389/fbioe.2026.1932282

**Published:** 2026-09-15

**Authors:** Tayyaba Yousaf, Ilyas Ahmad, Zohaib Younas, Donald Fernandes, Zia Ur Rehman Mashwani

**Affiliations:** 1 Department of Botany, Arid Agriculture University, Rawalpindi, Pakistan; 2 Department of Chemistry and Biology, Toronto Metropolitan University, Toronto, ON, Canada; 3 Pakistan Academy of Sciences, Islamabad, Pakistan

**Keywords:** clinical translation, glycemia, insulin, nanotechnology, novel drug delivery

## Abstract

Diabetes mellitus continues to be a global public health concern, owing to its increasing incidence, complex etiology, and the limitations of standard therapy regimens. Conventional therapeutic strategies often face several limitations including poor bioavailability, frequent administration, and absence of real-time blood sugar regulation. Recent advancement of smart nanotechnology has provided new opportunities for prevention, diagnoses, and management of diabetes as a part of smart health concept and precision medicine. This review highlights the recent trends in the design and application of nanotechnology for managing diabetes specifically focusing on two areas, glucose monitoring and drug delivery. Novel drug delivery systems are revolutionizing diabetes treatment, providing better pharmacokinetic control, adherence of patients, reduction of side effects, and target/stimuli responsive therapy. Recent developments in this area include sustained release implants and depots for extended periods of glycemic control; nanoparticles and liposomes for improved oral absorption and protection of insulin against enzymatic hydrolysis; transdermal and microneedles for minimal invasive delivery of insulin and peptides; inhalable dosage forms for faster systemic absorption; and glucose responsive ‘smart’ devices which deliver drugs only when required due to hyperglycemia. The use of nanotechnology-based biosensors and wearable devices has enabled the development of glucose monitoring based on their improved sensitivity, specificity, and integration into digital healthcare applications. Nanotechnology, coupled with artificial intelligence, and real-world health data, enhances personalized diabetes management through predictive monitoring and adaptive insulin delivery systems. Despite tremendous achievements, still some concerns persist, related to clinical translation, biosafety issues, and regulations. With continued interdisciplinary research that integrates the materials science, bioengineering, and clinical medicine, smart nanotechnology holds substantial promise to shift diabetes care, enabling an improvement in diabetic treatment from symptomatic to a physiologically sounder approach.

## Graphical Abstract

**Figure:**
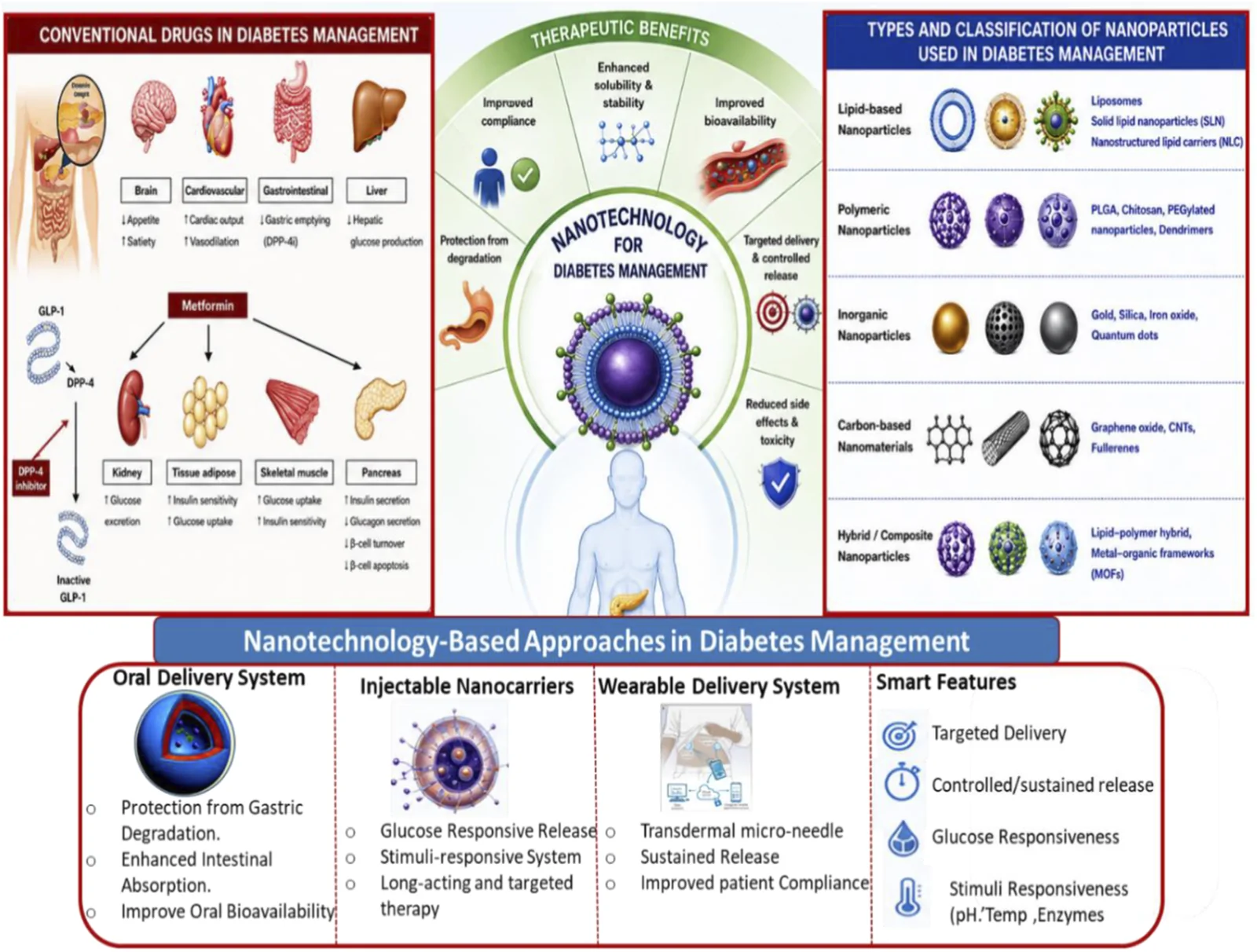

## 1 Introduction

Diabetes mellitus (DM) is a global metabolic disorder which is marked by long-term dysregulation of glucose homeostasis arising from poor insulin release, ineffective insulin functioning or both. Even with advancements in treatment over many years, diabetes continues to be one of the main causes of cardiovascular diseases, kidney failure, nerve damage, blindness and poor wound healing among others imposing healthcare burden worldwide (Gopalakrishnan et al., 2026). The underlying cause behind this predisposition may be the dysfunction of the pancreatic beta cells, or it may simply be due to a poor response of the body to insulin (El-Shaibany et al., 2026). Diabetes mellitus, as stated by the WHO (World Health Organization) and the international diabetes federation (IDF), is a major health problem on a global scale of the 21st century. Diabetes mellitus is the fourth most common chronic illness discovered after hypertension, arthritis, and dyslipidemia. Diabetes is a major cause of macro- and micro-vascular complications (Mohan et al., 2014). The burden of DM can be seen from the fact that there is an exponential growth in the population suffering from the disease and rising frequency of early mortality (El-Shaibany et al., 2026). In 2009, the prevalence of the diabetic population for both type-1 diabetes mellitus and type 2-diabetes mellitus (T1DM and T2DM) was about 285 million. This number later rose to 366 million in 2011, 382 million in 2013, 415 million in 2015, 425 million in 2017, 463 million in 2019, and 537 million in 2021. The prediction made by the IDF shows an increment to 643 million in 2030 and 783 million in 2045 accounting for an almost 46% increase (Saeedi et al., 2019; Miranda et al., 2024). Moreover, T2DM is responsible for up to 90% of all cases of diabetes. It is estimated that one in eleven individuals in the world suffer from T2DM, highlighting the urgent need for effective management strategies for diabetes management (Ahmed et al., 2018).

Pharmacological treatments and either continuous or intermittent glucose monitoring are the mainstays of modern diabetic therapy. Despite the fact that these methods have significantly improved clinical outcomes, there are still a number of important drawbacks. These include patient-dependent dose choices, delayed feedback control, non-physiological drug exposure profiles, and the intrusive character of many monitoring methods. Specifically, the spatiotemporal accuracy of endogenous pancreatic β-cell function cannot be dynamically replicated by exogenous insulin therapy. Patients are therefore still susceptible to long-term metabolic issues, hypoglycemia, and glycemic unpredictability (Halberg et al., 2019). Improving overall health and minimizing the effects of chronic complications are still goals, even though glycemic control is thought to be the initial step in managing diabetes. Diabetes mellitus is mostly treated with single-drug therapy, which includes insulin and insulin secretagogues, biguanides, insulin sensitizers, alpha glucosidase inhibitors, incretin mimetics, amylin antagonists, and sodium-glucose co-transporter-2 (SGLT2) inhibitors as shown in Figure 1 (Dahiru and Samuel, 2022). These medications have a wide range of adverse effects; their modes of action are also important considerations when administering them.

**FIGURE 1:**
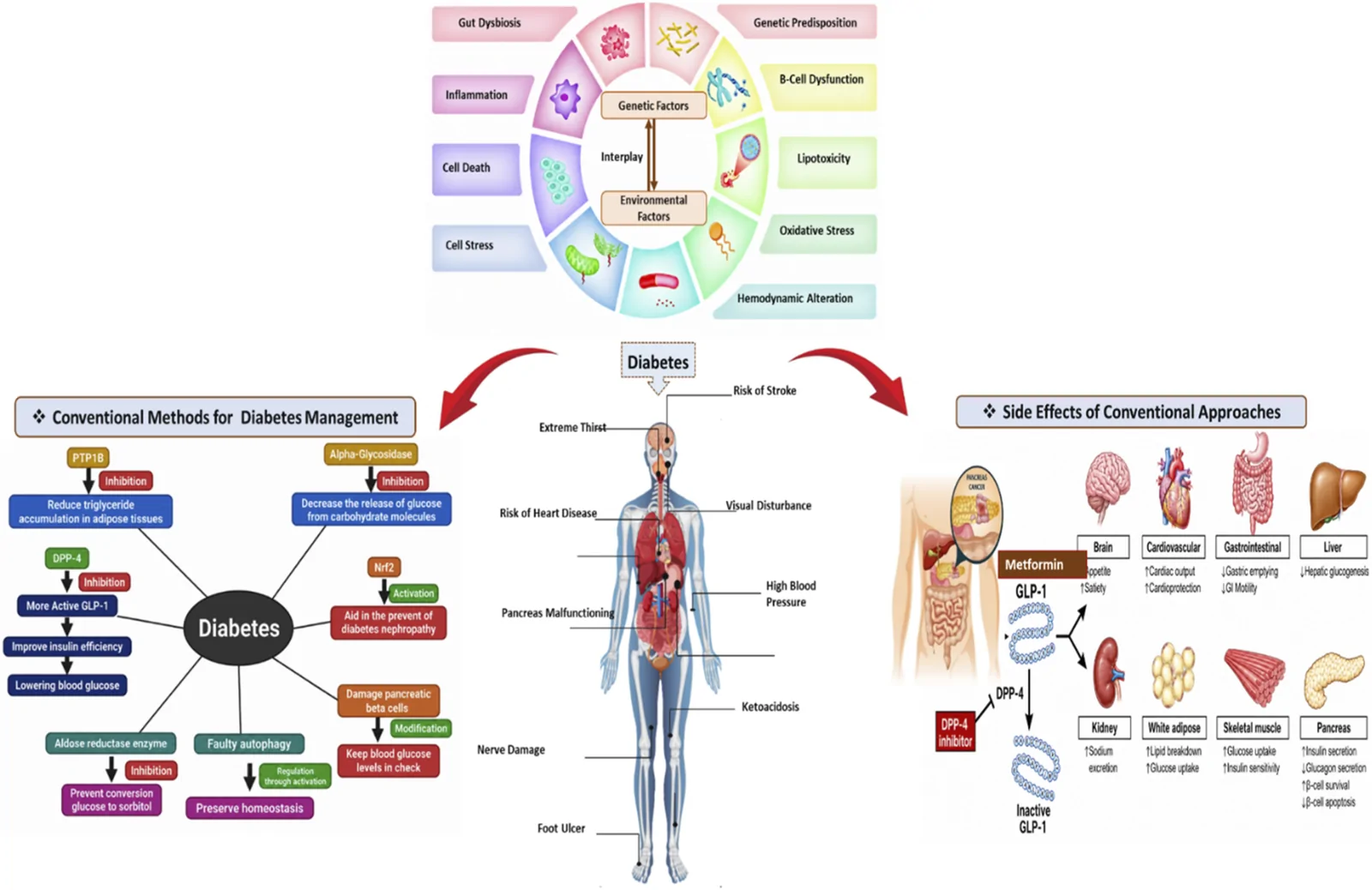
Schematic illustration of factors causing diabetes and management through conventional methods and their side effects.

Insulin therapy is commonly associated with hypoglycemia and weight gain, whereas metformin may cause gastrointestinal disturbances, including nausea, diarrhea, and abdominal discomfort, with rare cases of lactic acidosis in predisposed individuals. Sulfonylureas are associated with an increased risk of hypoglycemia and weight gain, while sodium-glucose cotransporter-2 (SGLT2) inhibitors have been linked to genital and urinary tract infections, volume depletion, and, in rare cases, diabetic ketoacidosis. Similarly, glucagon-like peptide-1 receptor agonists (GLP-1 RAs) frequently cause gastrointestinal adverse effects, including nausea and vomiting, which may affect treatment adherence (Jha et al., 2023). These limitations underscore the need for advanced therapeutic strategies capable of improving drug bioavailability, target specificity, and therapeutic efficacy while minimizing systemic toxicity, thereby highlighting the potential of nanostructure-based drug delivery systems in diabetes management.

A novel class of intelligent therapeutic and diagnostic materials that can detect and react to biochemical stimuli within the diabetic milieu has been made possible by recent developments in nanotechnology. The term “smart nanomaterials” refers to nanoscale systems that can recognize particular physiological signals and then produce an adaptive response in the context of managing diabetes. In reaction to environmental stimuli like glucose concentration, pH changes, or enzymatic activity, these reactions could include regulated medication release, sensor output modulation, or diagnostic function activation. It is crucial to differentiate this idea from a number of comparable nanotechnological approaches.

In this review, the term ‘smart’ relates mostly to stimuli-responsive nanomaterials which are able to detect physiological signals, specifically changes in glucose concentration and respond by giving feedback like controlled insulin secretion. This term establishes a conceptual basis for discussing the working principles of nano-based systems used in detection and glucose monitoring of diabetes. With the development of nanoscale carriers that can react to variations in glucose levels, pH gradients, oxidative stress, or enzyme activities, the prospects of self-regulated insulin delivery in addition to minimally-invasive glucose monitoring are finally on the horizon. The development of smart nanomaterials, such as polymers, lipids, inorganics, and hybrids, provides novel ways for drug release, bio-interface interaction, and signal transduction at the physiological length scale (Kaushal et al., 2025).

Nanotechnology has emerged as a promising strategy for improving both the diagnosis and treatment of diabetes through the development of highly sensitive glucose biosensors, targeted drug delivery systems, and glucose-responsive therapeutic platforms. Gold nanoparticles, graphene-based nanocomposites, and metal oxide nanostructures have demonstrated remarkable improvements in glucose detection sensitivity and real-time monitoring performance, while organic nanoparticles and nanocarriers have enhanced the bioavailability, stability, and controlled release of insulin and other antidiabetic agents. However, despite these encouraging advances, most nano-enabled systems remain at the preclinical or early clinical stage because challenges related to reproducible large-scale manufacturing, regulatory approval pathways, and comprehensive long-term biosafety evaluation have not yet been fully resolved which highlight the need for a critical and integrated assessment of recent innovations and their clinical prospects.

In this review, a comprehensive overview of recent innovations in smart nanomaterials for glucose-responsive drug delivery and metabolic sensing is presented. Particular emphasis is placed on the integration of these functionalities into emerging closed-loop diabetes management systems. While previous reviews have primarily focused on individual aspects of nanotechnology-based diabetes management, this review provides a comparative analysis of polymeric, lipid, inorganic, and composite nanoarchitectures with respect to glucose responsiveness, drug release characteristics, sensing performance, biosafety, and translational potential. Furthermore, it examines how the integration of nanostructured biomaterials with wearable electronics and artificial intelligence is accelerating the transition from glucose monitoring to autonomous diabetes management. By bridging nanomaterial design with clinical and technological translation, this review offers a comprehensive perspective on recent advances, current challenges, and future opportunities for the development of next-generation nano-enabled therapies for diabetes.

## 2 Methodology

To find pertinent research on smart nanomaterials for diabetes management, a structured literature search was carried out. Using keyword combinations such as “smart nanomaterials,” “nanotechnology in diabetes,” “glucose-responsive insulin delivery,” “nanocarriers,” “glucose biosensors,” and “wearable glucose monitoring,” peer-reviewed publications were gathered from PubMed, Scopus, Web of Science, and Google Scholar. In order to capture both fundamental and current advancements in nano-enabled therapeutic and diagnostic systems, publications from 2013 to 2026 were mainly taken into consideration. Retrieval articles were screened based on their titles, abstracts, and full texts, and duplicate records were excluded before detailed evaluation. Publications were selected according to predefined eligibility criteria, including original peer-reviewed research articles, mechanistic studies, *in vitro* and *in vivo* investigations, clinical studies, and high-quality review articles that provided significant scientific insight into nanostructure-enabled biosensing, therapeutic delivery systems, theranostic platforms, and emerging nanomedicine strategies for diabetes. Data extracted from the selected studies included the type and composition of nanostructures, synthesis and fabrication approaches, physicochemical characteristics, sensing mechanisms, therapeutic delivery strategies, biological targets, experimental models, diagnostic performance, therapeutic efficacy, biocompatibility, safety profiles, limitations, and translational potential. We took into account both experimental and review studies that had translational relevance, performance evaluation, or mechanistic insights. The type of nanomaterial, physicochemical characteristics, responsiveness mechanisms, drug release behavior, sensing performance, biosafety profiles, and translational development stage were among the important details taken from particular investigations. To illustrate the functional and clinically relevant distinctions across polymeric, lipid-based, inorganic, and hybrid nanomaterial platforms, the data were synthesized thematically and arranged into comparative tables for better understanding. A rigorous approach to literature identification, study selection, and critical evidence synthesis was adopted to ensure the reliability, transparency, and comprehensiveness of the findings presented in this review.

## 3 Conventional treatment methods and challenges in diabetes management

Despite the market being inundated with a large number of antidiabetic medications today, none of them cure diabetes completely. Each one carries its own deficiencies and side effects. One major problem is that the right amount of drug often cannot reach the target area in the body, because the drug breaks down too easily inside the harsh chemical environment of the body. Controlling blood glucose is the main goal in treating type 2 diabetes. Poor glucose control raises the risk of small and large blood vessel damage, and it adds to death rates, illness, and healthcare costs. Lifestyle changes are usually the first step, but most patients eventually need medicines as well. This happens because type 2 diabetes slowly destroys and weakens the insulin-producing beta cells in the pancreas, so blood sugar control keeps getting worse over time (Schroeder, 2026). Standard ways of drug delivery come with real risks while low blood sugar (hypoglycemia) is a prominent danger. On top of that, many drugs cause stomach irritation, diarrhea, dissolve poorly in water, leave the body quickly, and frequent dosing leads to the poor bioavailability of these pharmacological medications (Alhalmi et al., 2018). Most frequently used antidiabetic drugs such as metformin, thiazolidinediones, incretins, pramlintide, and alpha-glucosidase inhibitors all carry serious side effects. SGLT2 inhibitors, for instance, have been linked to heart failure. Large-scale heart-related clinical trials have shown that some diabetes drug classes actually protect against atherosclerotic heart disease, congestive heart failure, chronic kidney disease, and related metabolic problems, as shown in Figure 2. However, a lot of attention in the present era is going toward nanoparticle-based tools, including glucose biosensors, using nanoscale materials such as polymers, carbon nanotubes, magnetic nanoparticles, and quantum dots. Nanotechnology has pushed diabetes research toward new tools that can track glucose levels continuously and in real time. It has reshaped biomedical research by opening new ways to build better drugs and diagnostic tools. Because of their special properties, nanoparticles offer real advantages: better compatibility with the body, the ability to target specific cells, easier cellular uptake, and improved bioavailability (Sarkhel et al., 2024).

**FIGURE 2:**
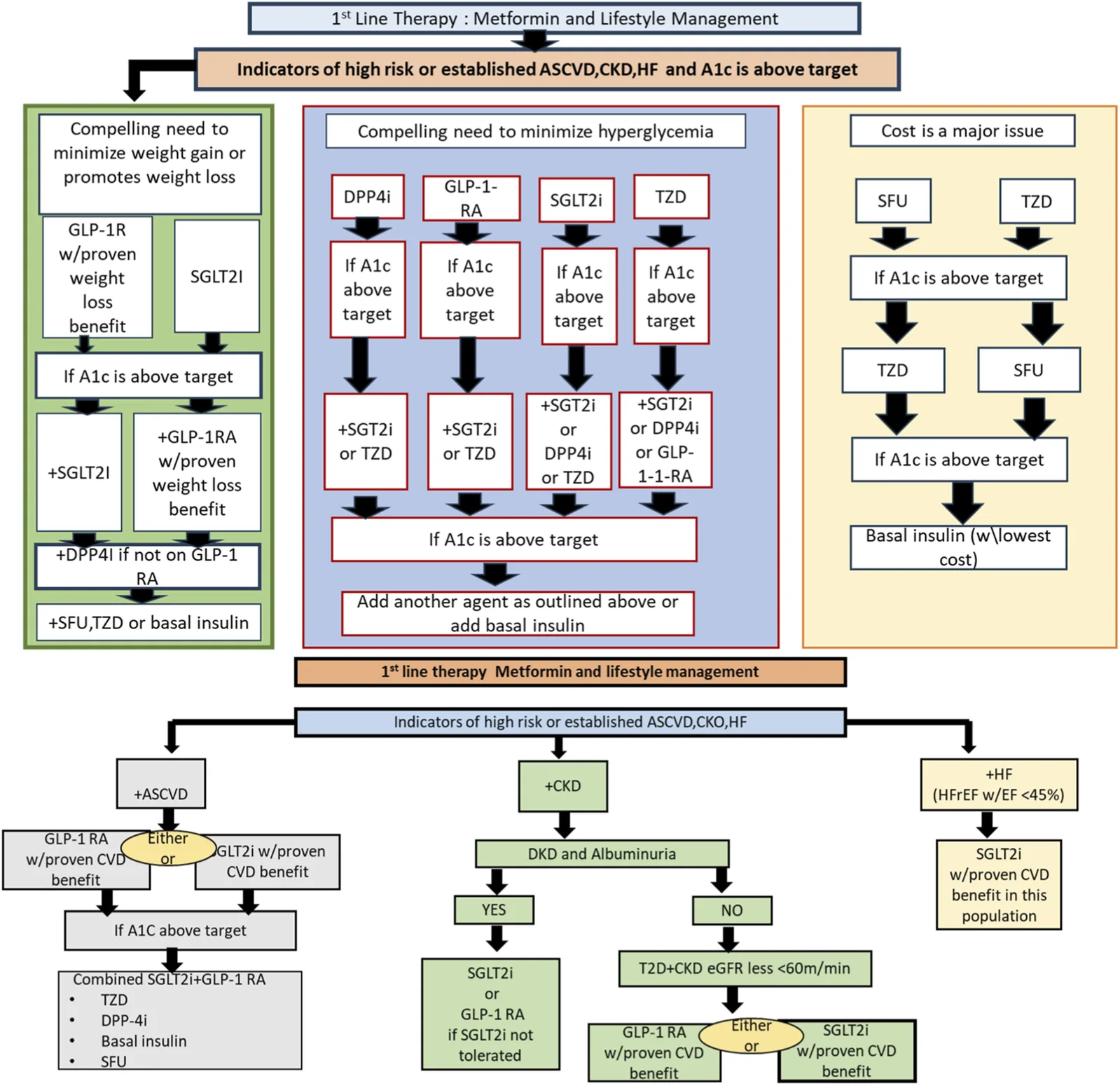
Pharmacological management of diabetes and risk factors.

## 4 Nanotechnology: a solution to conventional drug therapy

One of the most innovative and cutting-edge technologies for administering nano-therapeutics in medication delivery for treating diabetes is nanotechnology. Nanocarriers built by using nanotechnology have changed pharmaceutical research, allowing scientists to precisely control the shape, particle size, surface features, and release rate of active drug ingredients at the exact site where they are needed, at the right dose (Alhalmi et al., 2018). Nanocarriers have been synthesized from a range of natural, organic, and inorganic materials, including polymers, metals, ceramics, and lipids, as shown in Figure 3. The active drugs are included with these nanocarriers either by trapping it inside, encapsulating it, or attaching it to the surface. The unique properties of these different nanocarriers is to reduce the drawback caused by conventional drug delivery systems (Sharma et al., 2024). Aspects of this technology that are gaining much attention are nanoparticles as carriers for oral insulin formulations. Glucose biosensors built with nanoscale materials such as polymers, carbon nanotubes, magnetic nanoparticles, and quantum dots have shown much higher sensitivity (Wang et al., 2024). Nanotechnology has revalorized in the field of diabetes management and shifted the development towards new technologies including continuous, real-time tracking of glucose levels, improving the accuracy of readings, and enhancing the degree of compliance with therapy among patients. Smart nanomaterials are now being designed to react to biological signals such as pH, temperature, reactive oxygen species, glucose levels, and enzyme activity. Their classification, how they respond, and how close they are to real-world use are summarized in Table 1.

**FIGURE 3:**
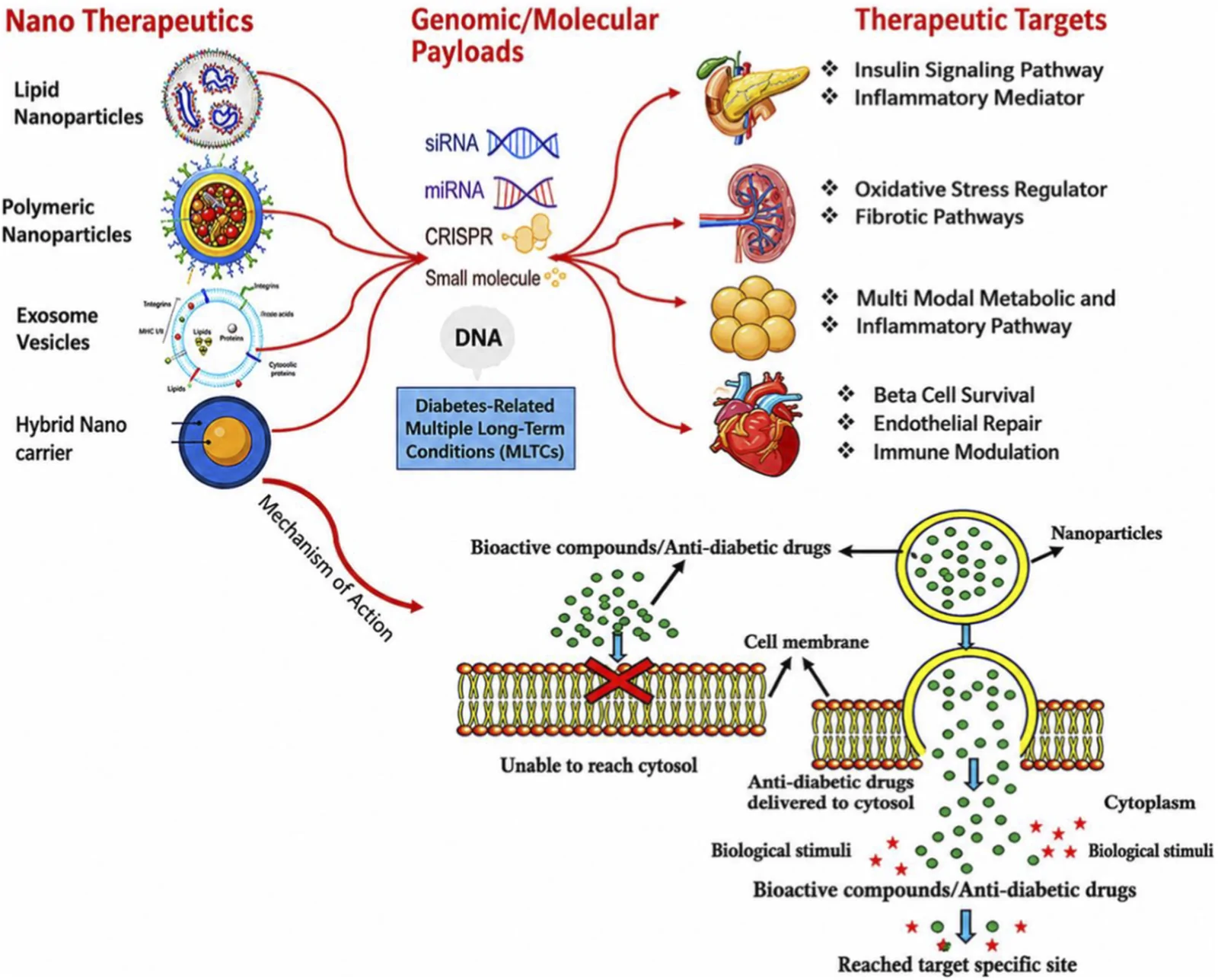
Nano therapeutics and their mechanism of action in diabetes management. Nano-therapeutic carriers encapsulate bioactive compounds, anti-diabetic drugs, or genomic/molecular payloads (siRNA, miRNA, CRISPR), enhancing cellular uptake and overcoming membrane barriers → delivering the therapeutic payload into the cytosol → targeting diabetes-related molecular pathways → modulating insulin signaling, inflammation, oxidative stress, fibrosis, metabolism, β-cell survival, and endothelial function → producing site-specific therapeutic effects and improving glucose homeostasis.

**TABLE 1:** Smart nanomaterials, their classification and mechanism of action along with toxicological profile and translational maturity.

Nanomaterial types	Size/structural features	Mechanism	Therapeutic control	Sensing performance	Toxicological profile	Translational maturity	Clinical relevance	References
Polymeric Nanoparticles (PLGA, PEG, Chitosan)	50–300 nm; zeta potential − 30 to +40 mV; drug loading ∼ 5–25 wt%; encapsulation efficiency 60%–95%	Stimuli-responsive systems triggered by glucose, pH, ROS, or enzymatic activity using functional groups such as phenylboronic acid or GOx-mediated reactions	Controlled and sustained insulin release ranging from 6 h to several days depending on polymer degradation rate and crosslink density	Moderate sensing when coupled with enzymatic systems; primarily designed for therapeutic delivery rather than sensing	Generally high biocompatibility and biodegradability; degradation products such as lactic and glycolic acid metabolized physiologically	Extensive preclinical development; polymeric carriers widely used in approved drug delivery systems	Promising platforms for glucose-responsive insulin delivery and long-acting formulations	(Sharma et al., 2022)
Lipid-based nanocarriers (Liposomes, SLNs, NLCs)	Particle size typically 80–400 nm; encapsulation efficiency ∼ 50–90%	Enzyme- or pH-responsive lipid membrane destabilization under hyperglycemia; glucose oxidase–mediated microenvironment changes	Release duration typically hours to days depending on lipid composition and bilayer stability	Limited intrinsic sensing; mainly used as drug delivery carriers	Very high biosafety due to lipid membrane similarity to biological membranes; metabolized through hepatic and lymphatic pathways	Preclinical to early clinical research; investigated for oral insulin and peptide therapeutics	High potential to improve oral bioavailability and pharmacokinetics of antidiabetic drugs	(Liu et al., 2025)
Metallic/Inorganic Nanomaterials (AuNPs, ZnO, Graphene, Silica)	Size typically 5–150 nm; mesoporous silica surface area ∼ 500–1,000 m^2^ g^−1^	Optical, electrochemical, or catalytic responsiveness to glucose concentration through enzyme immobilization or direct oxidation reactions	Generally not designed for drug release, though surface functionalization can enable triggered release	Very high sensitivity; detection limits in the nM–µM range with response times of seconds to minutes	Biosafety varies; potential risks include ROS generation, metal ion release, and organ accumulation depending on surface coating	Primarily preclinical and prototype-stage sensing platforms	Highly relevant for continuous glucose monitoring and wearable biosensors	(Alam et al., 2023)
Hybrid Nanostructures (Microneedles, Nano-hydrogels, Lipid–Polymer Hybrids)	Microneedle length 300–800 μm; hydrogel swelling ratio 200%–1,000%	Multi-stimuli responsive systems triggered by glucose, pH, temperature, or enzymatic activity	On-demand insulin delivery with >80% release efficiency under hyperglycemic conditions; rapid response within minutes	High sensing capability when integrated with embedded nanosensors or enzyme systems	Biosafety depends on composite materials; biodegradable polymer-based hybrids show favorable safety profiles	Advanced preclinical stage with emerging wearable prototypes	Strong potential for closed-loop therapeutic systems combining sensing and insulin delivery	(Zhang and Wu, 2023)

### 4.1 Polymeric nanoparticles

Biodegradable and biocompatible poly (lactic-co-glycolic acid) (PLGA) is a known polymer in pharma and biomedical sciences. Due to its flexibility and biocompatibility it has found application in the field of pharmaceuticals for designing drug delivery systems, bioengineering of tissues, and fabrication of medical devices. Drug delivery mechanisms research has shown that PLGA is perfectly suited for applications for controlled and sustained release in regulated settings. They include proteins, peptides, nucleic acids, and hydrophilic and hydrophobic drugs and can all be encapsulated with it. Antibiotics, vaccination, and anticancer medications are incorporated and released by nanoparticles and microparticles (Guo et al., 2023). PLGA-based nanoparticles and microparticles are now being synthesized, specifically for sustained and controlled release of insulin. Figure 4 illustrates the synthesis of poly (lactic-co-glycolic acid) (PLGA) through the copolymerization of lactic acid and glycolic acid while Figure 4B shows the drug release from PLGA nanoparticles occurs through sequential polymer hydration, drug diffusion, and polymer erosion, resulting in sustained release. Some PLGA microspheres are designed to reduce the injection frequency as insulin enabled over a full week or even a month. Other systems go further, using pH-sensitive or glucose-reactive components so that insulin is released only when blood sugar actually rises (Ghitman et al., 2020).

**FIGURE 4:**
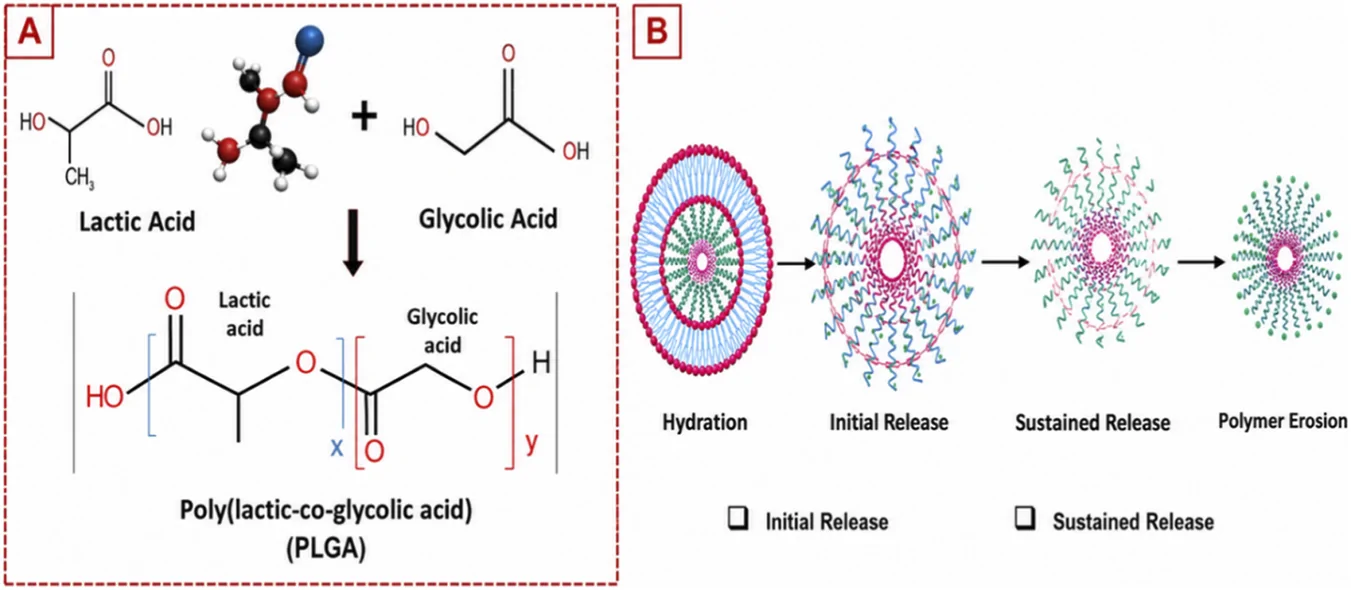
PLGA-based polymeric nanoparticles for controlled drug delivery. **(A)** Schematic illustration of the synthesis of poly (lactic-co-glycolic acid) (PLGA) through the copolymerization of lactic acid and glycolic acid. **(B)** Schematic representation of the controlled drug release mechanism from PLGA nanoparticles, showing polymer hydration, initial drug diffusion, and polymer erosion leading to sustained drug release.

Besides poly (lactic-co-glycolic acid) (PLGA), several biodegradable polymeric nanocarriers, including chitosan, alginate, poly (lactic acid) (PLA), polycaprolactone (PCL), polyethylene glycol (PEG)-based copolymers, and poly (β-amino esters), have been extensively investigated for the delivery of antidiabetic therapeutics owing to their excellent biocompatibility, tunable degradation profiles, and capacity for surface functionalization. Chitosan and alginate exhibit intrinsic mucoadhesive and glucose-responsive properties that are particularly advantageous for oral insulin delivery, whereas PCL provides prolonged drug release due to its slower degradation kinetics. PEGylated polymers further improve systemic circulation, reduce protein adsorption, and enhance nanoparticle stability (Danhier et al., 2012). However, PLGA remains the most extensively investigated and clinically translated biodegradable polymer because of its established safety profile, regulatory approval, predictable hydrolytic degradation into metabolically biocompatible lactic and glycolic acid, high encapsulation efficiency, and the ability to achieve sustained and controlled drug release (Kaur et al., 2022). Despite these advantages, PLGA-based systems may exhibit an initial burst release, local acidification caused by degradation products, and limited loading efficiency for certain hydrophilic biomolecules. Consequently, recent research has increasingly focused on hybrid polymeric formulations and surface-engineered PLGA nanoparticles to overcome these limitations while preserving their favorable translational characteristics (Makadia and Siegel, 2011).

### 4.2 Metallic and inorganic nanomaterials

Metallic and inorganic nanomaterials are gaining more attention in diabetes care because of their distinct physical and chemical properties, including strong electrical conductivity, optical response, catalytic ability, and adjustable surface chemistry at the nanoscale level. These qualities make them well suited for highly sensitive glucose biosensors, signal boosting, and the development of implantable or wearable continuous glucose monitoring (CGM) devices. At the nanoscale, their sensing ability depends mainly on surface chemical reactions, how fast electrons move, and quantum confinement effects, all of which boost signal strength and detection sensitivity far beyond what bulk materials can achieve. Some inorganic nanoparticles are also being studied as carriers for insulin as well as tools to help to regenerate pancreatic beta cells, manage oxidative stress, and heal wounds linked to diabetes complications (Cardoso et al., 2025). The main types used in diabetes nanotechnology include gold nanoparticles, graphene and other carbon-based structures, zinc oxide and other metal oxide nanoparticles, mesoporous silica nanoparticles, and quantum dots (Luo et al., 2025). Gold nanoparticles attached to glucose-sensitive linkers or polymer coatings in drug delivery systems have shown great potential for triggered insulin release. Their plasmonic properties also allow them to be used in photothermal systems, where under external stimuli, localized heating (for example, from near-infrared light) speeds up drug release. An *in situ* sensor can be built by combining a molecularly imprinted polymer with gold nanoparticles (MIP-AgNPs) on a screen-printed electrode for achieving selective, enzyme-free glucose detection across a wide range (1.0 μM–1.0 mM), with very low detection limits, thanks to glucose being pre-concentrated by the imprinted polymer. Integrated gold nanoparticles also lower the glucose oxidation potential and reduce interference from substances like ascorbic acid, giving accurate glucose readings in blood serum through combined electrochemical methods (Cardoso et al., 2025). Among metallic nanoparticles, gold and silver nanoparticles (AgNPs) stand out the most in therapeutic research. Silver nanoparticles show strong conductivity, chemical stability, and catalytic power, along with notable anti-inflammatory, antimicrobial, and antidiabetic effects. Research shows that silver nanoparticle systems can fight diabetes broadly, work against drug-resistant infections, and cause very little toxicity to the body (Husain et al., 2023). Studies on silver nanoparticles in diabetes care have focused mainly on how well they can improve the delivery of antidiabetic drugs. For example, combining silver nanoparticles with insulin or other glucose-lowering drugs can make those drugs more stable and more available in the body. Evidence also suggests that silver nanoparticles can help control blood glucose levels better and safeguard the pancreatic beta cells from oxidative stress, both of which matter a great deal in diabetes management (Wahab et al., 2022).

Metal oxide nanoparticles, especially zinc oxide, titanium dioxide, and iron oxide, are also strong candidates for biosensing because of their catalytic activity, semiconductor behavior, and compatibility with enzymes. Zinc oxide nanoparticles, in particular, improve enzyme attachment and electron transfer, making them useful for enzyme-free glucose sensors. This happens due to high density surface defects and semiconductor properties that speed up charge transfer between enzymes and electrodes, which improves glucose oxidation and strengthens the detection signal. In another study, the drug empagliflozin was successfully attached onto zinc oxide nanoparticles measuring about 17 nm, with the attachment pattern best explained by the Freundlich isotherm model, confirming effective surface binding (Tripathy and Kim, 2018).

### 4.3 Hybrid and smart responsive nanomaterials

The most sophisticated class of smart nanosystems for managing diabetes are hybrid and stimuli-responsive nanomaterials, which combine many functional materials for achieving dynamic responsiveness to physiological inputs. These materials are built to sense shifts in glucose, pH, temperature, or enzyme activity, and to adjust drug release or monitoring signals accordingly. Their design mimics the endogenous endocrine function of pancreatic β-cells, progressing toward closed-loop and autonomous diabetes regulation. Hybrid nanostructures may bring together organic polymers, lipid layers, inorganic nanoparticles, biological enzymes, and smart molecular switches. This combination gives better mechanical strength, higher bioavailability, longer circulation time in the body, and combined effects which are not achieved by a single-material nanoparticle alone (Harun-Ur-Rashid and Jahan, 2024). Glucose-responsive hybrid nanosystems mark a major step forward in diabetes treatment, because they release insulin only when blood sugar is high. These systems typically rely on one or more glucose-sensing methods: enzyme-based response using glucose oxidase, reversible binding chemistry using phenylboronic acid, or glucose recognition through lectins such as concanavalin A. By redesigning the chemical bonding between phenylboronic acid and diol groups to reduce their binding strength, researchers found that glucose can compete more effectively with the network at normal body concentrations, making the hydrogel respond better to glucose. Tests measuring gel softness and insulin release confirmed that networks with weaker phenylboronic acid-diol binding released insulin more reliably in response to glucose, compared to older chemical designs (Xian et al., 2025). Glucose oxidase-based nanosystems (GOx) create local acidity and hydrogen peroxide when glucose levels rise, which causes the surrounding polymer structure to break down or swell, releasing insulin in proportion to the glucose present. Hybrid systems combining glucose oxidase with PLGA, lipids, or hydrogels have shown responses that closely match how the body’s own hormone feedback systems work (Liao et al., 2025).

Hybrid nanomaterials have also been built into microneedle patches, as shown in Figure 5, which illustrates how a glucose-responsive microneedle patch delivers insulin. The diagram shows the functional mechanism of the glucose-sensitive nanocarriers built into a microneedle patch for delivering insulin through the skin. Insulin and glucose oxidase are packed together inside glucose-responsive particles through a self-assembly process. When glucose levels rise, the glucose oxidase reacts with glucose to produce hydrogen peroxide, which breaks apart the nanocarrier structure. Once the patch is placed on the skin, glucose moves into these particles and triggers insulin release. These dual-sensitive particles respond to glucose while minimizing inflammation responses as compared to systems that do not respond to hydrogen peroxide, leading to safer and more effective insulin delivery through the skin.

**FIGURE 5:**
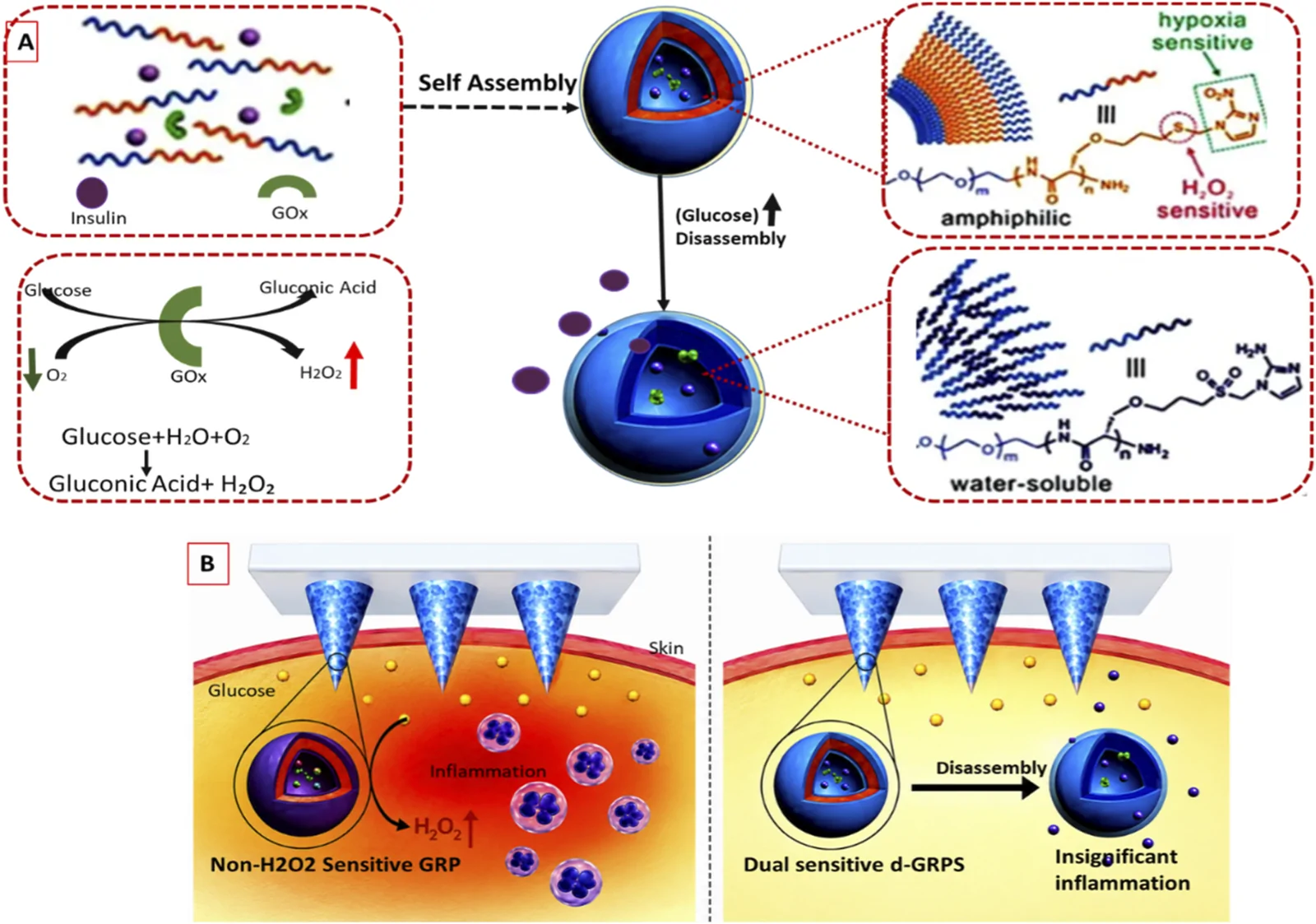
Schematic illustration of a glucose-responsive microneedle patch for insulin delivery. **(A)** Fabrication and glucose‐responsive self‐assembly of insulin‐loaded nanoparticles: glucose oxidase (GOx)‐mediated glucose oxidation generates gluconic acid and H_2_O_2_, inducing nanoparticle disassembly through changes in the amphiphilic structure. **(B)** Mechanism of glucose‐responsive insulin delivery *in vivo*, under hyperglycemic conditions, with elevated glucose triggering GOx-mediated H_2_O_2_ generation and nanoparticle disassembly, promoting insulin release and reducing glucose levels. Under normoglycemic conditions, minimal nanoparticle disassembly occurs, resulting in negligible inflammation and controlled insulin release.

## 5 Conventional glucose monitoring and core limitations

Diabetes and related metabolic disorders are becoming more common among people, leading to the urgent need for glucose monitoring devices. Diagnosis and monitoring depend on different tools. HbA1C (Glycated hemoglobin) tests provide an indication of average blood glucose levels during 3 months but lack real time data (Pullano et al., 2022). Self-monitoring of blood glucose, recommended at least three times a day for patients on intensive insulin therapy, uses finger-prick meters to guide treatment decisions. Continuous glucose monitoring (CGM) is a major step forward, tracking glucose around the clock through a sensor placed under the skin, paired with a transmitter and a receiver or smartphone app. CGM lowers the risk of low blood sugar episodes, reduces HbA1c levels, and gives real-time insight into glucose trends (Bender et al., 2025). However, these methods also have drawbacks leading to major health concerns. Finger-prick testing causes pain, carries infection risk, and can produce sampling errors. CGM devices may need regular calibration, can drift out of accuracy over time, and sometimes still require a finger-prick test to confirm readings (Min et al., 2025). So, shifting towards the advanced glucose biosensor is the actual need to combat these drawbacks.

### 5.1 Advanced glucose biosensors: clinical need and emerging opportunities

The rapidly increasing global burden of diabetes has intensified the demand for glucose monitoring technologies capable of providing accurate, continuous, and real-time assessment of glycemic fluctuations to facilitate timely therapeutic intervention and improve long-term metabolic control. Although conventional self-monitoring of blood glucose (SMBG) and continuous glucose monitoring (CGM) systems have substantially improved diabetes management, they remain constrained by limitations including invasiveness, frequent calibration requirements, physiological lag between blood and interstitial glucose, limited operational lifetime, biofouling, and reduced analytical accuracy under dynamic physiological conditions (Benjamin, 2002). Furthermore, these systems primarily monitor glucose concentrations without offering predictive insights or integration with personalized therapeutic strategies. Consequently, there is an urgent clinical need for next-generation glucose biosensors that combine high analytical performance with improved patient comfort, continuous operation, and compatibility with intelligent diabetes management platforms.

### 5.2 Glucose biosensing from first generation to smart sensor

Glucose biosensors have played a central role in diabetes management since the very first enzyme electrode was developed. Over time, they have become more sensitive, more selective and easier to access moving from early lab prototypes to miniaturized commercially available devices. Modern biosensors can now measure glucose not just in blood, but also in interstitial fluid and other body fluids such as sweat and tears, allowing real-time, continuous, and often non-invasive monitoring that supports better diabetes care (Teymourian et al., 2020). The evolution of glucose biosensors can be grouped into several generations based on how they detect glucose, as shown in Figure 6.

**FIGURE 6:**
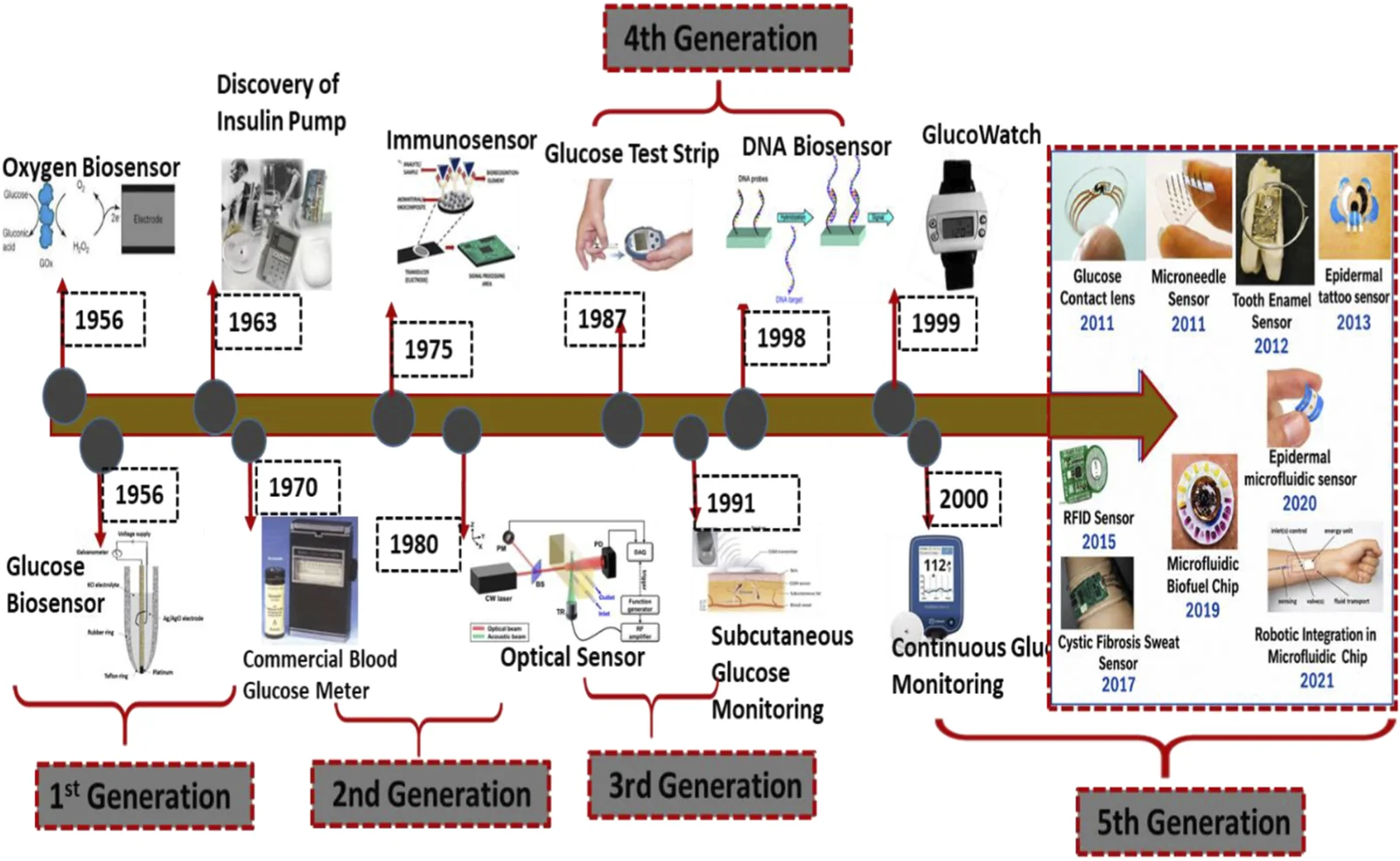
Generation wise evolution of glucose biosensors, from first generation to fifth generation. The evolution of glucose monitoring has progressed from early glucose biosensors and blood glucose meters to optical, subcutaneous, and continuous glucose monitoring technologies. Recent fifth-generation systems incorporate minimally invasive and wearable platforms, enabling more convenient, continuous, and personalized glucose monitoring.

Glucose biosensor technology has gone through five distinct generations, each one solving problems left behind by the one before it. The first generation began with the original glucose biosensor built on the Clark oxygen electrode, alongside the discovery of immobilized enzymes. These early devices depended on the natural oxygen use of glucose oxidase reactions, but they were limited by their dependence on oxygen and by interference from other electrically active substances (Malik et al., 2023). The second generation solved these problems using mediator-based biosensors, which allowed electron transfer without needing oxygen. This period saw the commercial launch of glucose biosensors for home blood sugar testing, along with the appearance of optical sensors in the 1980s, setting the stage for portable home-use glucose meters. The third generation, spanning the 1980s and 1990s, introduced direct electron transfer between the enzyme and the electrode improving both accuracy and reliability. Glucose test strips, introduced in 1987 made testing far more convenient and portable while the early 1990s brought the first ideas around subcutaneous glucose monitoring paving the way for less invasive devices. The fourth generation, covering the 2000s and 2010s, brought nanotechnology and DNA-based sensing into the picture. Major milestones included DNA-based sensors and devices like the GlucoWatch (1998–1999), followed by continuous glucose monitoring systems in the early 2000s. As nanobiosensors expanded, detection sensitivity improved significantly with miniaturization of devices. From 2011 onward, a new generation of wearable platforms emerged, including glucose-sensing contact lenses (2011), microneedle sensors (2011), tooth enamel tattoo sensors (2012), epidermal tattoo sensors (2013), RFID-based sensors (2015), sweat sensors for cystic fibrosis (2017), microfluidic biofuel chips (2018), epidermal microfluidic sensors (2020), and robotic integration within microfluidic chips (2021). The fifth generation marks a shift toward smart, non-invasive, and fully integrated biosensing systems (Rajamohan and Sun, 2025). Depending on the type of nanomaterial composition and sensing strategy, recent electrochemical glucose biosensors have achieved sensitivities ranging from 22.3 to over 17,000 μA mM^-1^cm^-2^, limits of detection as low as 1.4 nM, linear detection ranges extending from 0.001 to 13 mM, and response times of less than 5 s. These improvements are attributed to the unique physicochemical properties of nanomaterials, including high specific surface area, excellent electrical conductivity, superior electrocatalytic activity, and versatile surface functionalization, which collectively enhance signal amplification, analytical accuracy, operational stability, and reproducibility (Ambaye et al., 2024). Consequently, nanotechnology-based glucose biosensors are increasingly recognized as promising platforms for real-time, continuous, and highly reliable glucose monitoring in precision diabetes management. Current research is focused on building wearable, implantable, and lab-on-chip systems that monitor glucose continuously and in real time while keeping patient discomfort to a minimum. These newer devices are designed to support personalized treatment plans and transmit data wirelessly without interruption. On top of that, combining artificial intelligence, the Internet of Things, and robotics with biosensing platforms is opening the door to predictive healthcare, where real-time data analysis does more than just track glucose changes. It can also anticipate dangerous blood sugar swings before they happen, allowing for more proactive and precise diabetes management.

## 6 Recent advances in glucose biosensors via smart nanotechnology

Nanotechnology has completely upheaved glucose sensing by providing new strategies to improve sensitivity response, by reducing detection limit, shortening response times, and enabling the development of miniaturized devices suitable for point-of-care and personalized healthcare. Different kinds of biomarkers have been introduced such as insulin and glycated hemoglobin (HbA1c) present in biological fluids, including blood, tear, and urine, and used for diabetes diagnosis. Blood glucose levels are detected by glycemic levels, even though blood glucose levels may not be a crucial marker for long-term glycemic management due to their considerable changes in daily foods, stress levels, and physical activity. HbA1c is associated with red blood cell’s lifespan and measured after two to 3 months. HbA1c levels connected to total hemoglobin (Hb) are less than 6% in the normal range, whereas values of more than 9% suggest poor glycemic management. The American diabetes association (ADA) has proposed these values as criteria for diabetes diagnosis (Sherwani et al., 2016). However other methods should also be introduced to assess diabetes in real time. Depending on the signal source, current nanomaterial-based biosensors for diabetes biomarkers primarily use electrochemical and optical detection (Lyons and Basu, 2012). Metal nanoparticles (e.g., gold, silver, platinum, nickel, copper) are widely used to enhance electrochemical signals and boost catalytic activity. Carbon-based nanostructures, including carbon nanotubes (CNTs), graphene, and graphene oxide, contribute high conductivity, chemical stability, and versatile scaffolds for enzyme or mediator immobilization as shown in Figure 7. Most recently, two-dimensional (2D) materials such as transition metal dichalcogenides (TMDs) and MXenes have provided opportunities for next-generation biosensors, leveraging their layered architectures, high electron mobility, and tunable surface chemistry. Overall, these nanotechnology-based innovations not only enhance the analytical performance of glucose biosensors but also provide the development of flexible, wearable, and non-invasive monitoring systems, aligning with the increasing demand for continuous glucose monitoring and personalized diabetes management.

**FIGURE 7:**
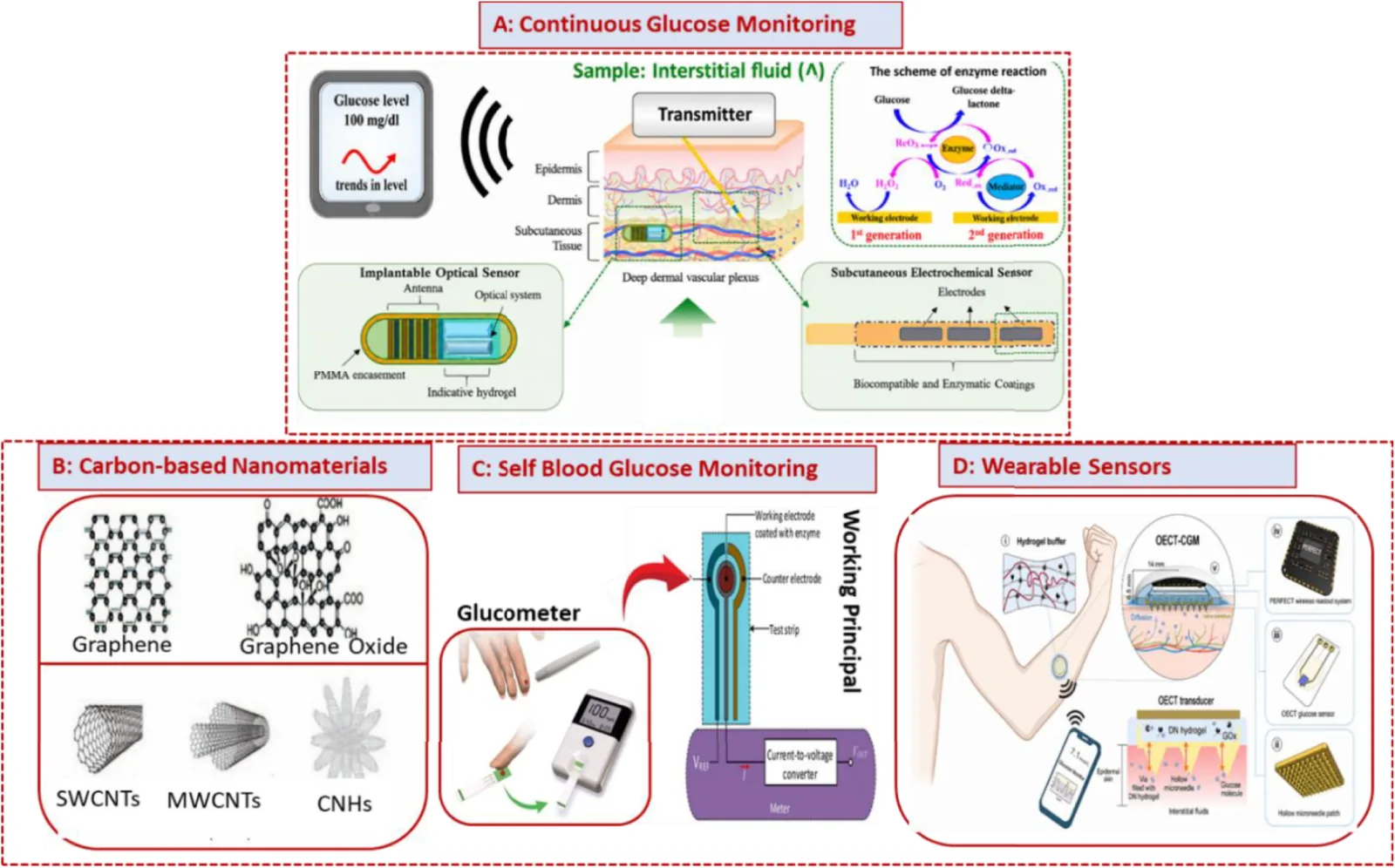
Recent advances in nanostructured biosensors in real time glucose detection and monitoring. **(A)** Illustration of advanced glucose monitoring through continuous sensing of interstitial glucose using implantable and enzymatic sensors. **(B)** Carbon-based nanomaterials enhance sensor performance. **(C)** Self-monitoring devices measure blood glucose levels through a simple finger-prick blood sample, providing rapid and convenient glucose readings for daily diabetes management. **(D)** Wearable glucose sensors enable continuous, minimally invasive monitoring of glucose levels, providing real-time data through compact and connected devices for personalized diabetes management.

### 6.1 Metal and metal oxide nanoparticles in glucose detection

The surface area of nanostructured metals is a key factor for glucose oxidation and can be increased for enhancing electrocatalysis and improving sensitivity and detection efficiency (Khan, 2017). Noble metal nanoparticles (Pt, Au, Ag) provide good conductivity, stability, and enzyme immobilization, boosting electron transfer in enzymatic sensors. Several studies provide basis for use of Ag NPs to increase glucose sensor performance. Ren et al. used Ag NPs to enhance direct electron transfer between the electrode and GOx, immobilizing GOx in silver sol and cross-linking with polyvinyl butyral in glutaraldehyde on a platinum electrode. This modification increased electron transfer rates, resulting in a steady-state response three times higher than GOx alone, as Ag NPs acted as conductive pathways between enzyme prosthetic groups and the electrode surface (Ren et al., 2005). Serra et al. leveraged the localized surface plasmon resonance (LSPR) of Ag NPs for optical glucose sensing. Ag NPs capped with polyvinyl alcohol showed a selective LSPR response only to glucose, while other sugars and buffers caused no change in absorbance, confirming specificity (Serra et al., 2009). Li et al. introduced an ATR fiber-optic glucose-based sensor with Ag NPs grown on its surface with a U shaped fiber of 0.25 cm radius and a CO_2_ laser light source. The Ag NP-modified ATR sensor provided threefold better results with higher sensitivity and resolution compared to conventional ATR sensors, measuring glucose absorption at multiple infrared wavelengths (1,037–1,081 cm^-1^) (Li et al., 2015). Gold nanoparticles (Au NPs) also significantly increased glucose biosensors by enhancing electrode surface area, improving catalytic activity, and facilitating efficient electron transfer, which allows higher GOx enzyme loading, greater sensitivity, and lower detection limits. Feng et al. developed a chitosan–AuNPs (CHI–Au NPs) nanocomposite on a glassy carbon electrode (GCE) *via* a simple electrodeposition method. The sensor showed a detection limit (LOD) of 3.70 × 10^−4^ M and linearity from 4.00 × 10^−4^ M to 1.07 × 10^−2^ M for glucose. Gold based nanoparticles anchored nitrogen-doped graphene (Au NP/NG) nanohybrids offered good stability, reproducibility and electrochemically active surface area, increasing electron transfer between the electrode and glucose (Feng et al., 2009). Jena and Raj designed a non-enzymatic glucose sensor using Au NPs self-assembled on a 3D silica network. In phosphate buffer (pH 9.2), Au NPs efficiently catalyzed glucose oxidation at a low potential (0.16 V) without redox mediators or enzymes. The catalytic activity increased with the Au NP surface area (Jena and Raj, 2006).

Platinum nanoparticles (Pt NPs) are widely used in glucose biosensing due to their outstanding performance and qualities which enhance sensitivity, stability, and selectivity. Various studies have combined Pt NPs with various nanomaterials to improve sensor performance. Zhai et al. developed a highly sensitive glucose enzyme sensor using Pt NPs-polyaniline (PA) hydrogel heterostructures. Pt NPs were uniformly loaded onto a 3D PA hydrogel matrix, combining the hydrogel’s conductivity and porosity with the catalytic activity of Pt NPs. The hydrogel facilitated high-density enzyme immobilization and substrate diffusion, while Pt NPs efficiently decomposed H_2_O_2_ generated during glucose oxidation. The sensor showed good performance with a sensitivity of 96.1 μA mM^-1^·cm^-2^, a fast response time of 3 s, a linear range of 0.01–8 mM, and a low LOD of 0.7 μM (Zhai et al., 2013). A Pt-decorated hollow carbon sphere (Pt/HCSs) electrode was developed and immobilized with GOx using Nafion. Pt NPs (≈2.29 nm) were well dispersed on HCSs, catalyzing the oxidation and reduction of enzymatically liberated H_2_O_2_. The sensor showed high sensitivity (4.1 μA mM^-1^), rapid response (<3 s), a low LOD (1.8 μM), and a wide linear range (0.04–8.62 mM). It showed high stability, reproducibility, and less interference from ascorbic acid and uric acid, achieving good recovery in blood serum samples (Luhana et al., 2012).

Nickel oxide nanoparticles (NiO NPs) have been extensively explored for both enzymatic and non-enzymatic glucose sensing due to their excellent electrocatalytic properties, high stability, and tunable nanostructures. NiO NPs were synthesized *via* an eco-friendly electro-exploding wire technique at 24, 36, and 48 V (Ahamad et al., 2024). The NiO 48V sample exhibited the smallest particle size, rugged surface, and highest Ni^3+^ content, enhancing redox currents and glucose oxidation in alkaline media (0.1 M NaOH, pH 13). This sensor achieved a wide linear range (0.1–1 mM), high sensitivity (1,202 μA mM^-1^·cm^-2^), and a low LOD of 0.25 μM, with excellent stability, reproducibility, repeatability, and selectivity. Ultra-small NiO NPs can also be prepared by calcination of nickel-p-phenylene diamine complexes (Venkadesh et al., 2021). NiO NPs were electrodeposited onto Santa Barbara Amorphous-15/multi-walled carbon nanotubes (SBA-15/MWCNT) modified carbon paste electrodes producing a non-enzymatic sensor with a linear range of 0.06–400 μM and LOD of 0.023 μM. The sensor demonstrated rapid response, high sensitivity, and potential for point-of-care applications. It was also reported that nickel oxide nanoparticles (NiO NPs) are highly effective for glucose sensing due to their excellent electrocatalytic activity, tunable nanostructures, and high stability (Baghayeri et al., 2017).

A non-enzymatic glucose sensor with excellent catalytic action, lower working potential (0.55 V), and quick response (∼3 s) was prepared from ZnO/Co_3_O_4_/rGO nano-composites through hydrothermal reaction. Three-dimensional hierarchical Co_3_O_4_ nanoflowers were created and contrasted with spherical and nanorod morphologies. The nanoflowers displayed outstanding electrocatalytic activity, attaining two linear ranges (5–60 μM and 0.2–3.0 mM) with great selectivity, sensitivities of 693.02 and 228.03 μA•mM^-1^•cm^-2^, and LODs of 0.04 and 0.14 μM, respectively because of their high surface-to-volume ratio (Kalkozova et al., 2025). The higher surface area and electrocatalytic activity of nanoflowers and activated carbon composites were shown to make them very effective for stable, sensitive, and selective glucose detection, with potential uses in wearable technology and point-of-care.

### 6.2 Electrochemical nanosensors

The most advanced class of glucose monitoring systems enabled by nanotechnology are electrochemical nanosensors. Carbon nanotubes (CNTs), carbon nanofibers, graphene and carbon quantum dots have emerged as important nanostructures for next-generation glucose sensors because of their excellent conductivity, biocompatibility, and exceptional surface area (Farrokhnia et al., 2022). Gluconic acid is created when electrochemical biosensors react with blood glucose. By determining the concentration of gluconic acid, by tracking the current signal, the blood glucose level may be found. Because of their exceptional biocompatibility, carbon-based luminescent materials have garnered a lot of interest in the construction of optical sensors for many applications. They have also been extensively researched and used in optoelectronics, photocatalysis, medical diagnostics, and biosensors. Because of this, a number of sensors that use nanomaterials have been reported to assess glucose levels, making testing easier, and increasing sensitivity (Hovancová et al., 2017). Glucose sensors have shown quick reaction times and great sensitivity for several analytes, based on variations in the fluorescence intensity of the luminous nanoparticles, all while being markedly straightforward in terms of design and manufacturing (Peng et al., 2012; Xie et al., 2016).

#### 6.2.1 Carbon nanotubes (CNTs)

The remarkable physicochemical and electrochemical characteristics of carbon-based nanomaterials have become essential elements in the creation of high-performance glucose sensors. The vast surface area of carbon nanotubes makes it possible to immobilize enzymes or directly adsorb glucose, increasing the number of active sites for glucose oxidation and enhancing sensor sensitivity. Rapid electron transfer between the electrode surface and redox-active species is made possible by their superior electrical conductivity, which is essential for attaining a quick and effective electrochemical reaction. Single-dimensional conductive networks, reduce resistance and significantly speed up charge transfer at the electrode interface, with carbon nanotubes (CNTs) being well known for their capacity to improve electron transfer kinetics. Because of their great surface area, superior conductivity, and mechanical durability, carbon nanotubes (CNTs) are frequently utilized in glucose sensors. This makes them perfect for usage as direct conductive materials for non-enzymatic sensing as well as scaffolds for enzyme immobilization. Highly densified CNT fibers (HD-CNTf) incorporated into a polymer matrix with open-ended CNTs exposed at the interface were used to create a CNT microelectrode set (CNT µ-ES) (Gupta et al., 2021). The Cu NPs/HD-CNTf microsensor demonstrated a very low LOD (28 nM), high sensitivity (1942 nA μM^-1^·cm^-2^), and no interference from physiological interferents in electrochemical experiments conducted in 0.1 M NaOH. CuO-modified CNTs that resemble fibers were created as wearable, flexible glucose sensors (Muqaddas et al., 2023). For a non-enzymatic glucose sensor, functionalized CNTs and Co(OH)_2_ show enhanced stability and repeatability. With a LOD of 43.2 μM and strong linearity throughout 50–700 μM glucose, the sensor confirmed the viability of affordable CNT-based sensors. Robust frameworks for enzyme immobilization are also offered by CNTs functionalized with certain groups. For example, selective enzymatic glucose detection was achieved using CNT nanoelectrode ensembles (NEEs) and a single-wall CNT field-effect transistor (FET) (Pourasl et al., 2014).

By virtue of its large surface area, conductivity, and mechanical resilience, CNTs improve glucose sensors. CuNP-based non-enzymatic sensors have high sensitivity, broad linear ranges, and low LODs. Effective enzymatic glucose detection is made possible by functionalized CNTs, which enhance enzyme immobilization, selectivity, and stability. Flexible, wearable, and reasonably priced glucose sensors are supported by CNT-based microelectrodes and NEEs, which enable interference-free detection without membranes or facilitators. In conclusion, CNT-based glucose sensors enable both enzymatic and non-enzymatic detection techniques by combining superior conductivity, surface area, mechanical durability, and functional adaptability. These platforms exhibit low LOD, broad linear ranges, great sensitivity, and compatibility with non-invasive, wearable, and versatile applications.

#### 6.2.2 Graphene and reduced graphene oxide (rGO)

The remarkable electrical conductivity, huge surface area, and high electrocatalytic activity of graphene and its derivatives, such as rGO, improve electron transport and sensor performance in glucose detection. Palladium oxide nanoparticles (PdO NPs) combined with rGO printed on a cellulose substrate were used to immobilize GOx. The biosensor successfully detected glucose in human serum with a limit of detection of 0.046 μM, sensitivity of 0.03239 μA/μM, and outstanding repeatability, stability, and specificity (Chen et al., 2024). For sweat glucose detection, Au-Pt alloy NPs and chitosan-GOx composites were added to microfabricated rGO electrodes on flexible polyimide. The sensor demonstrated a linear range of 0–2.4 mM, sensitivity of 48 μA/mM⋅cm^2^, LOD of 5 μM, and quick response (∼20 s) (Xuan et al., 2018). In a different investigation, a foam nickel electrode treated with GO improved the detection sensitivity and current response in a Pt/Ag/AgCl system. The sensor showed excellent linearity (*R* ^2^ = 0.9998), a LOD of 13.3 μM, and a more environmentally friendly method of measuring blood glucose level (Chang et al., 2020). For both enzymatic and non-enzymatic glucose sensors, graphene-based materials, particularly rGO, offer superior platforms. These sensors can be used for wearable applications as well as blood and sweat glucose monitoring. To improve performance, they can be coupled with metal nanoparticles (PdO, Au-Pt, Cu(II)) or three-dimensional nanostructures.

#### 6.2.3 Carbon quantum dots (CQDs)

The peculiar optical, electrical, and catalytic properties of carbon quantum dots (CQDs) and their derivatives, including graphene quantum dots (GQDs) and nitrogen-doped CQDs (N-CQDs), make them attractive platforms for both electrochemical and optical glucose sensing. Rajmane et al. (2025) developed graphene quantum dots (GQDs) through a low-temperature hydrothermal approach using citric acid and investigated their fluorescence-based response toward glucose. The sensor exhibited a linear detection range of 2.0 × 10^-5^–2.0 × 10^−4^ M and a limit of detection (LOD) of 1.53 × 10^−5^ M. The glucose–GQD interaction was further evaluated using Langmuir adsorption analysis, from which a binding constant (K_b_) of 4.05 × 10^5^ was reported. In this context, K_b_ ​ represents the Langmuir adsorption/binding constant and reflects the extent of glucose interaction with the GQD surface (Rajmane et al., 2025). The analytical applicability of the sensor was further evaluated using spiked human urine samples. The reported recovery values were 95%, 99%, and 97%, demonstrating good agreement between the added and measured glucose concentrations in the tested urine samples (Wang et al., 2019). However, this evaluation represents *ex vivo* validation in a biological matrix rather than continuous wearable or implantable glucose monitoring.

### 6.3 Wearable and implantable nano devices

Nanomaterial-based wearable and implanted sensing devices have made significant progress toward closed-loop, minimally invasive, and therapeutically translatable monitoring models. This includes the skin-adhesive and sweat-based sensors and implantable smart biosensing platforms, which are currently in trend. Transdermal or exocrine monitoring without blood collection is made possible by sweat-activated biosensors, ultrathin epidermal patches, and versatile nanostructured electrodes used in microneedle arrays. Real-time glycemic responsiveness coordinated with smartphone-based analytics is demonstrated by graphene-based electronic tattoos and PBA-functionalized hydrogels (Min et al., 2023). In order to demonstrate its potential for ongoing health management applications, Özkahraman et al. created a black phosphorus/graphitic carbon nitride (BP/g-CN) heterostructure incorporated into a wearable skin patch that allows real-time, non-invasive sweat glucose monitoring with enhanced electrocatalytic activity and dependable wireless data transmission (Özkahraman et al., 2025).

PEG-modified carbon nanotube (CNT) microelectrode fibers and biodegradable metal-polymer composites are some examples of implantable nanosensors that have shown extended operational durability while reducing foreign-body immune reactions. These platforms provide personalized digital metabolic profile and progress the creation of autonomous insulin delivery devices when combined with wireless monitoring (Hossain et al., 2025). Moreover, a battery-independent implantable glucose sensor based on typical electrical impedance spectroscopy demonstrated a strong correlation between bioimpedance signals and reference blood glucose levels across a wide physiological range (77.4–523.8 mg dL^-1^), when tested *via* subcutaneous implantation in a porcine model. This highlights the viability of long-term, energy-efficient continuous glucose tracking without the need for repeated blood sampling or harmful tissue reactions (Ollmar et al., 2023).

### 6.4 Comparative evaluation and future perspectives of smart nanomaterial-based glucose biosensors

Despite the remarkable progress achieved with individual nanomaterials, each class exhibits distinct advantages and inherent limitations that influence its suitability for glucose biosensing applications. Noble metal nanoparticles, such as gold and platinum, provide excellent electrocatalytic activity and high analytical sensitivity but are associated with high production costs and limited large-scale applicability. In contrast, transition metal-based nanomaterials, including nickel and copper nanoparticles, offer cost-effective alternatives with favorable catalytic properties, although their long-term stability and selectivity may be affected by the sensing environment (Ambaye et al., 2024). Carbon-based nanomaterials, including graphene and carbon nanotubes, possess exceptional electrical conductivity, large specific surface area, and excellent biomolecule immobilization capability; however, challenges related to material aggregation, reproducible fabrication, and large-scale production remain. More recently, two-dimensional materials such as MXenes and transition metal dichalcogenides have demonstrated enhanced electron-transfer kinetics and versatile surface chemistry, making them attractive candidates for next-generation biosensors, although further investigation of their long-term biocompatibility and clinical performance is still required. Therefore, current research is increasingly focused on hybrid nanocomposites that combine the complementary properties of different nanomaterials to achieve improved sensitivity, selectivity, stability, flexibility, and clinical translatability for advanced glucose biosensing (Ma et al., 2025).

## 7 Current trends in novel drug delivery systems (NDDS) for diabetes mellitus

Recent developments in nanotechnology have resulted in the creation of novel NDDS strategies intended to address the shortcomings of traditional diabetes treatments. In order to improve glycemic control, reduce side effects, and increase patient compliance, these systems concentrate on regulated, sustained, and targeted medication delivery.

### 7.1 Oral insulin delivery systems

A significant development in the treatment of diabetes is oral insulin administration, which provides a non-invasive substitute for subcutaneous injections. Because of enzymatic breakdown and low intestinal epithelial permeability, conventional insulin is poorly absorbed in the gastrointestinal tract. To get over these obstacles, new drug delivery techniques have been created, such as liposomes, polymeric micelles, mucoadhesive systems, and carriers based on nanoparticles. These technologies improve intestinal absorption, offer regulated release, and shield insulin from enzymatic breakdown, all of which increase bioavailability. Furthermore, certain oral insulin formulations are being designed to react to blood glucose levels, allowing for more physiological blood glucose management. Oral insulin delivery systems have great potential to improve patient adherence, lessen injection-related discomfort, and transform diabetes treatment, even if they are still mostly in the developmental or early clinical trial stage (Zhao et al., 2020).

### 7.2 Nanoparticle drug delivery systems

By providing targeted, prolonged, and controlled medication release, nanoparticle-based drug delivery systems have completely transformed contemporary therapies. These systems are made up of materials including polymers, lipids, metals, or biodegradable compounds, with their particles usually ranging in size from 10 to 1,000 nm. Nanoparticles are being investigated for enhancing the pharmacokinetic profile, stability, and bioavailability of insulin and other antidiabetic medications in the treatment of diabetes mellitus (Souto et al., 2019). The small size of nanoparticles allows them to pass through biological barriers efficiently, keeping the encapsulated medicine from degradation and permitting controlled release at the target region. To improve intestine absorption and extend the therapeutic impact, for example, polymeric nanoparticles like PLGA poly (lactic-co-glycolic acid) and chitosan-based nanoparticles have been investigated for oral insulin administration. Conversely, glucose-responsive medication delivery systems that release insulin in relation to blood glucose levels have been demonstrated to be possible using lipid and gold nanoparticles. Nevertheless, there are still certain restrictions, such as possible cytotoxicity, intricate production procedures, and difficulties in large-scale manufacturing. Despite these, using nanotechnology in diabetes treatment offers a promising way to address the drawbacks of traditional insulin delivery methods, providing more accurate glucose control and improved patient compliance (Almohmadi et al., 2025). Nanoparticles-based drug delivery systems exhibit distinct physicochemical and biological properties that determine their therapeutic performance. Polymeric nanoparticles, particularly PLGA and chitosan-based systems, offer biodegradability, protection of protein therapeutics, and tunable release kinetics, whereas lipid-based nanoparticles provide high biocompatibility and efficient encapsulation of diverse therapeutic agents. Inorganic nanoparticles, such as gold-based systems, offer unique surface properties and stimulus-responsive drug release, but their long-term accumulation and biosafety require further consideration. Therefore, the selection of a nanoparticle platform should be guided by drug stability, loading capacity, release kinetics, biological compatibility, administration route, and translational feasibility.

### 7.3 Transdermal drug delivery systems in diabetes mellitus

One of the most promising and patient-friendly methods for managing diabetes mellitus is the use of transdermal drug delivery systems (TDDS). By delivering medications directly into the systemic circulation through the skin, these methods circumvent gastrointestinal breakdown and hepatic first-pass metabolism. Compared to traditional subcutaneous insulin injections, this method offers a number of benefits, including increased patient compliance, painless administration, regulated release, and stable plasma drug concentrations (Ng and Gupta, 2020). However, the stratum corneum, which prevents big macromolecules like insulin from penetrating, makes the skin a major barrier. Numerous cutting-edge transdermal methods, such as microneedles, iontophoresis, electroporation, and ultrasound-mediated administration, have been developed to get around this restriction. Among these, microneedle-based patches have drawn particular interest for the treatment of diabetes. These microneedles, which are composed of biodegradable or dissolving polymers, gently pierce the stratum corneum to release insulin into the dermal layer, where it is taken up by the bloodstream. Glucose-responsive microneedle patches, which can detect increased blood glucose levels and release insulin in response, are the result of recent advances. These devices reduce the risk of hypoglycemia by automatically controlling insulin release by sensing mechanisms based on glucose oxidase or phenylboronic acid (Islam et al., 2021). Furthermore, wearable smart insulin patches with feedback-controlled pumps and continuous glucose monitoring (CGM) sensors are being developed to enable real time insulin regulation akin to that of an artificial pancreas. Using lipid-based vesicular carriers in transdermal formulations, such as transferosomes and ectosomes, is another interesting strategy. By changing lipid packing and boosting membrane fluidity, these ultra-deformable vesicles improve medication penetration through the skin. Such carriers can greatly increase the transdermal permeability and bioavailability of insulin and other antidiabetic medications, according to recent studies. Additionally, transdermal systems that integrate polymeric films, gels, and nanocarriers enable sustained and steady release profiles, lowering the frequency of doses and enhancing long-term glycemic control.

### 7.4 Implantable and smart drug delivery systems

Wearable and smart drug delivery systems have garnered a lot of attention because of their capacity to react dynamically to physiological changes, especially glucose fluctuations. Implantable systems are tiny devices or biodegradable depots placed under the skin or within body tissues that continuously release insulin or other antidiabetic agents over extended periods of time. These systems help maintain consistent plasma glucose levels while lowering the frequency of injections and improving patient adherence. These systems mimic the action of pancreatic β-cells by using stimuli-responsive polymers that release insulin automatically when blood glucose levels rise. Glucose-sensitive polymers, hydrogels, or nanoparticles containing glucose oxidase, phenylboronic acid, or concanavalin assessment components are frequently the foundation of smart delivery systems. These materials experience structural alterations that cause the release of insulin when high glucose concentrations are detected. This ensures accurate glycemic control and reduces the danger of hypoglycemia. Furthermore, artificial pancreases have been developed as a result of the integration of wireless communication technologies with microelectronic sensors. These intelligent devices provide a near-physiological approach to diabetes treatment by autonomously regulating insulin supply through feedback control and continuously monitoring blood glucose levels. Implantable and smart delivery systems, while still in the early phases of development, have enormous potential to transform diabetes care by offering patient-friendly, responsive, and long-lasting management choices that improve quality of life and therapeutic efficacy (Jaiswal et al., 2025). Working mechanism of an implantable drug delivery system is shown in Figure 8.

**FIGURE 8:**
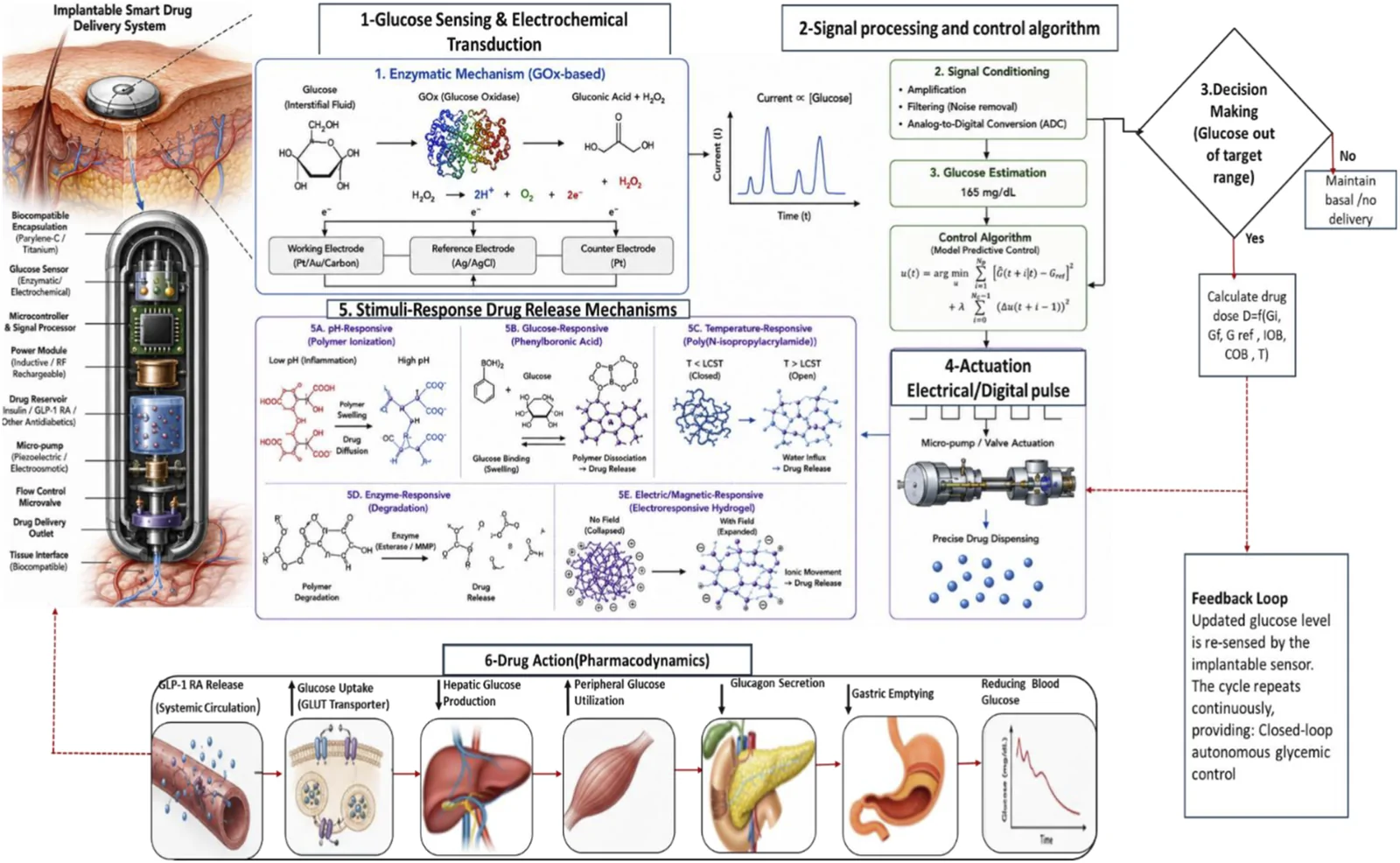
Mechanistic view of implantable glucose-responsive drug delivery system: (1) Glucose oxidase (GOx)-based electrochemical sensing continuously measures interstitial glucose concentration. (2) Control algorithm processes the sensor signal and calculates the precise therapeutic dose in real time. (3) Stimuli-responsive nanomaterials (pH-, glucose-, temperature-, enzyme-, or magnetic-responsive) trigger controlled drug release via a micro-pump. (4) Drug delivery actuation. (5) Released insulin/GLP-1 restores glucose homeostasis, while a closed-loop feedback system continuously adjusts drug delivery according to changing glucose levels. (6) Drug action and pharmacodynamics response: The released drug regulates glucose through insulin-mediated uptake, hepatic and peripheral glucose utilization, glucagon regulation, and gastric emptying. The resulting glucose changes are fed back to the sensing system for continuous, adaptive glycemic control.

## 8 Artificial intelligence (AI) and digital health in nanostructure-based diabetes management

The integration of artificial intelligence (AI), digital health technologies, and nanostructure-based platforms is rapidly transforming diabetes management by enabling intelligent, data-driven, and personalized healthcare solutions. AI-assisted continuous glucose monitoring (CGM) systems employ machine learning algorithms to analyze real-time glucose dynamics, predict glycemic excursions, optimize insulin dosing, and facilitate individualized clinical decision-making, thereby improving glycemic control and reducing the risk of hypoglycemic events (Bajramagic et al., 2026). Furthermore, the convergence of wearable biosensors, Internet of Medical Things (IoMT) technologies, smartphone-based digital therapeutics, and cloud computing enables continuous remote monitoring, seamless data integration, and timely therapeutic interventions, supporting a transition toward precision diabetes management (Wavhule, 2026). Advances in nanotechnology have further accelerated this paradigm by integrating smart nanomaterials with glucose-responsive drug delivery systems and highly sensitive biosensing platforms, allowing simultaneous glucose detection and controlled therapeutic release. These multifunctional nanoplatforms provide enhanced sensitivity, targeted drug delivery, improved therapeutic efficacy, and reduced systemic adverse effects compared with conventional treatment strategies. The future integration of AI-driven predictive analytics with glucose-responsive nanocarriers and closed-loop insulin delivery systems is expected to enable autonomous therapeutic platforms capable of real-time glucose sensing, adaptive insulin administration, and personalized disease management. Nevertheless, successful clinical implementation will require overcoming several challenges, including data interoperability, cybersecurity and patient-data privacy, algorithm validation, regulatory approval, large-scale manufacturing, and long-term biosafety evaluation of multifunctional nanostructures before widespread clinical adoption (Gopalakrishnan et al., 2026).

## 9 Advantages, future prospects, and challenges of NDDS in diabetes management

By addressing the shortcomings of traditional medications, novel drug delivery systems (NDDS) have demonstrated impressive potential in improving diabetes care. They offer improved diabetes management with enhanced bioavailability, targeted drug administration, and prolonged release, which can decrease side effects and lower dose frequency. Techniques including transdermal patches, nano-formulations, and implantable devices improve patient comfort and compliance, particularly for those who need long-term insulin or oral hypoglycemic medication. In the future, the integration of smart technologies, such as wearable and glucose-responsive delivery systems, promises individualized, real-time medication administration that is adapted to changing blood glucose levels. These developments may improve therapeutic results and lessen the problems brought on by long-term hyperglycemia. Nevertheless, there are a number of obstacles to the widespread use of NDDS in the treatment of diabetes. Large-scale manufacturing and commercialization may be constrained by high production costs, intricate fabrication procedures, and strict regulatory constraints. Long-term stability, possible toxicity of new carriers, and inconsistent patient reactions continue to be major concerns. Accessibility may be limited by the need for surgical intervention or specific monitoring for some advanced delivery systems, such as implanted or glucose-responsive devices. Furthermore, it is challenging to thoroughly assess the safety and effectiveness of emerging NDDS therapies due to a lack of clinical data. Despite of remarkable advances in nanostructure-based technologies for diabetes, their clinical translation remains limited by several critical challenges. The successful commercialization of these platforms requires standardized regulatory frameworks, scalable manufacturing processes with consistent batch-to-batch quality, and comprehensive long-term biosafety evaluations to establish their pharmacokinetic profile, biodegradability, and potential immunotoxicity. Addressing these translational barriers through harmonized regulatory guidelines and robust clinical validation will be essential for the successful integration of nanostructure-based systems into routine diabetes care.

## 10 Conclusion

Smart nanomaterials are revolutionizing the landscape of diabetes management through enhanced glucose detection, dynamic delivery of insulin, and use in novel closed-loop treatment methods. Research involving nanomaterials and novel drug delivery systems including those that are polymeric, lipids, inorganic, and hybrids showcase ways in which nanotechnology can help control the stability, release rate, and accuracy of sensing and interface interaction of insulin. The use of glucose-sensitive polymers, electrocatalysis, fluorescent and plasmonic nanosensors, and microneedles as platforms for delivering drugs demonstrates the shift from passive to active drug delivery mechanisms. Fluorescent and plasmonic nanosensors, and microneedle-based delivery platforms highlight the transition from conventional passive drug delivery toward dynamically regulated, biomimetic control of glycemia. NDDS reduces side effects and dosage frequency in addition to improving therapeutic results. Despite these advantages, before they are widely used in clinical settings, issues related to large-scale production, regulatory approval, cost-effectiveness, and long-term safety must be resolved. Personalized and automated diabetes management systems may be made possible by future research that integrates nanotechnology, biosensors, and artificial intelligence. However, NDDS has enormous potential to transform diabetes care by moving the emphasis from standard insulin delivery to more effective, patient-friendly, and precision-based treatment methods.
